# Systemic Sclerosis 2011

**DOI:** 10.1155/2011/308231

**Published:** 2012-04-09

**Authors:** Lorinda Chung, Oliver Distler, Laura Hummers, Eswar Krishnan, Virginia Steen

**Affiliations:** ^1^Division of Immunology and Rheumatology, Stanford University School of Medicine and Palo Alto VA Health Care System, 3801 Miranda Avenue, Palo Alto, CA 94305, USA; ^2^Department of Rheumatology, University Hospital Zurich, Gloria street 25, 8091 Zurich, Switzerland; ^3^Division of Rheumatology, Johns Hopkins University, 5501 Hopkins Bayview Circle, Room 1B.7, Baltimore, MD 21224, USA; ^4^Division of Immunology and Rheumatology, Stanford University School of Medicine, 1000 Welch Road, Suite 203, Palo Alto, CA 94305, USA; ^5^Division of Rheumatology, Georgetown University, 3800 Reservoir Road, NW, Pasquerilla Healthcare Center, Suite 3004, Washington, DC 20007, USA

Systemic sclerosis (SSc) is a systemic autoimmune disease characterized by widespread fibrosis affecting the skin, internal organs, and vasculature. However, there are currently no systemic, disease-modifying therapies available for the treatment of the overall condition, and the outcomes remain poor. Studies into disease pathogenesis have identified several pathways that are dysregulated in SSc, and novel targeted therapies are currently being developed. In this special issue, we invited authors to submit original research articles, review articles, or case reports/case series describing preclinical, translational, or clinical studies related to new therapies for SSc.

A set of papers in this special issue focuses on identification of new therapeutic targets in preclinical and translational studies. V. J. Moulin's paper is a review article describing the role of apoptosis in the initiation and maintenance of disease in SSc, through effects on the immune system, vascular damage, and fibroblast proliferation. This report discusses potential therapies targeting apoptosis and the Fas/FasL pathway that could be investigated in patients with SSc. Other paper describes vascular changes in the bleomycin-induced mouse model of SSc. The authors discuss the use of this mouse model to investigate targeting fibrosis, apoptosis, and cellular adhesion molecules for the treatment of vascular disease in SSc. The paper by T. Radstake et al. reviews the evidence that hypoxia contributes to the pathogenesis of SSc, with a focus on the role of hypoxia inducible factor (HIF)-1 alpha in the vasculopathy, immune dysregulation, and fibrosis in SSc. This paper summarizes potential therapeutic interventions to bypass the dysfunctional hypoxic pathway in SSc. The paper by R. De Vries et al. describes an original research study evaluating the accumulation of advanced glycation end products (AGE) in the skin of patients with SSc compared with controls using a technique called skin autofluorescence. Although this study did not find a significant difference in AGE accumulation in SSc skin compared with control samples, use of angiotensin-converting enzyme inhibitors and angiotensin II receptor blockers in the SSc patients may have confounded the results. The paper by E. G. Kroon et al. is an original research article evaluating the expression of Types I and III interferons (IFNs) and interferon-stimulated genes (ISG) in peripheral blood mononuclear cells from SSc patients compared with controls. This study confirmed the increased basal expression of Type I IFNs and the ISG 2′5′OAS in SSc, but found no induction of Type III IFNs. This paper provides further evidence that targeting the IFN pathway may be useful in the treatment of SSc. The paper by S. M. Violette et al. reviews the preclinical data supporting the role of the integrin *α*v*β*6 in the activation of fibrosis via the transforming growth factor (TGF)-*β* pathway. This paper summarizes in vivo evidence of the utility of blocking *α*v*β*6 for the treatment of lung fibrosis and provides rationale for pursuing this therapeutic approach in patients with SSc-associated interstitial lung disease.

Another set of articles includes reviews of novel therapies that are currently being evaluated for the treatment of SSc. The paper by M. Anderson et al. reviews the role of interleukin-6 (IL-6) in SSc, summarizing evidence of effects on B cells, inflammation, fibrogenesis, and endothelial cell activation. The paper reviews the rationale for ongoing clinical trials of agents blocking IL-6 transsignaling for the treatment of SSc. The paper by S.-N. Liossis et al. reviews the published literature supporting the role of B cells in SSc, summarizing data from animal models and human studies. The authors then review the results of four clinical trials assessing the effects of B cell depletion with rituximab therapy on skin disease and lung function in patients with SSc. The paper by R. F. Spiera and J. Gordon reviews the preclinical and clinical studies of tyrosine kinase inhibitors, with a focus on experience with imatinib, in the treatment of SSc and related fibrotic conditions. The authors conclude that interpretation of the results from the completed proof-of-concept studies is difficult due to the small size and heterogeneity of the populations studied and the open-label designs. The review article by K. Phillips et al. describes published studies investigating the utility of phosphodiesterase-5 inhibitors (PDE-5-I) in the treatment of Raynaud's phenomenon (RP) and/or digital ulcers (DU), detailing results of studies using sildenafil, vardenafil, and tadalafil. The authors also list the ongoing clinical studies of PDE-5-I for RP and DU. The paper by D. F. Fiorentino et al. reviews the role of endothelin-1 in the pathogenesis of SSc-associated vascular disease and summarizes the published reports evaluating the use of endothelin receptor antagonists in the treatment of RP and DU.

One of the sets in this special issue includes two articles describing rare clinical manifestations of SSc, gastric antral vascular ectasias and primary biliary cirrhosis, and management strategies for these entities. The paper B. Markewitz et al. is a case series describing the tolerability and efficacy of naltrexone for the treatment of pruritus and gastrointestinal symptoms in three patients with SSc. The paper by K. Nikolov and M. Baleva reviews the rationale for using intravenous immunoglobulins (IVIG) in the treatment of SSc. The authors also summarize the published case reports and series supporting the potential efficacy of IVIG in the treatment of skin sclerosis in SSc.

In summary, this special issue provides an interesting compilation of articles addressing potential emerging therapies for the treatment of SSc, including information gleaned from preclinical, translational, and clinical studies. We, the guest editors, hope readers find that the manuscripts included herein offer a comprehensive summary of the current status of drug development and promising therapies for SSc.

*Lorinda Chung* *Lorinda Chung* *Oliver Distler* *Oliver Distler* *Laura Hummers* *Laura Hummers* *Eswar Krishnan* *Eswar Krishnan* *Virginia Steen* *Virginia Steen*

